# The plant hormone ethylene promotes abiotic stress tolerance in the liverwort *Marchantia polymorpha*

**DOI:** 10.3389/fpls.2022.998267

**Published:** 2022-10-18

**Authors:** Priyanka S. Bharadwaj, Lizbeth Sanchez, Dongdong Li, Divine Enyi, Bram Van de Poel, Caren Chang

**Affiliations:** ^1^ Department of Cell Biology and Molecular Genetics, University of Maryland, College Park, MD, United States; ^2^ Division of Crop Biotechnics, Department of Biosystems, University of Leuven, Leuven, Belgium; ^3^ KU Leuven Plant Institute, University of Leuven, Leuven, Belgium

**Keywords:** abiotic stress, ethylene, liverwort, heat stress, salinity, nutrient deficiency, far-red (FR) light

## Abstract

Plants are often faced with an array of adverse environmental conditions and must respond appropriately to grow and develop. In angiosperms, the plant hormone ethylene is known to play a protective role in responses to abiotic stress. Here we investigated whether ethylene mediates resistance to abiotic stress in the liverwort *Marchantia polymorpha*, one of the most distant land plant relatives of angiosperms. Using existing *M. polymorpha* knockout mutants of Mp*ein3*, and Mp*ctr1*, two genes in the ethylene signaling pathway, we examined responses to heat, salinity, nutrient deficiency, and continuous far-red light. The Mp*ein3* and Mp*ctr1* mutants were previously shown to confer ethylene insensitivity and constitutive ethylene responses, respectively. Using mild or sub-lethal doses of each stress treatment, we found that Mp*ctr1* mutants displayed stress resilience similar to or greater than the wild type. In contrast, Mp*ein3* mutants showed less resilience than the wild type. Consistent with ethylene being a stress hormone, we demonstrated that ethylene production is enhanced by each stress treatment. These results suggest that ethylene plays a role in protecting against abiotic stress in *M. polymorpha*, and that ethylene has likely been conserved as a stress hormone since before the evolutionary divergence of bryophytes from the land plant lineage approximately 450 Ma.

## 1 Introduction

Environmental stresses, such as high temperature, salinity, nutrient deficiency, drought, and flooding, can gravely impact plant growth and development. Plants have evolved an ability to deal with these stresses using hormone signaling networks that help maintain growth and development (Verma et al., 2016). In angiosperms, one of the plant hormones that mediates responses to biotic and abiotic stresses is ethylene gas, which affects many aspects of growth and development (Abeles et al., 1992; Dubois et al., 2018; Chen et al., 2022). For example, plants synthesize ethylene in response to abiotic stress [e.g., under flooding (Fukao and Bailey-Serres, 2008), iron deficiency (Romera et al., 1999), or high salinity (Achard et al., 2006)]. The protective role of ethylene signaling has been shown using ethylene mutants. For example, ethylene biosynthesis mutants in petunia are more sensitive to salt stress (Naing et al., 2022), as are tobacco ethylene-insensitive mutants (Cao et al., 2007). In *Arabidopsis thaliana*, ethylene-insensitive mutants are less resistant to heat stress (Larkindale and Knight, 2002), have reduced basal thermotolerance (Huang et al., 2021), and are defective in shade avoidance (i.e., a low ratio of Red : Far-Red light) (Pierik et al., 2009).

The thalloid liverwort *Marchantia polymorpha* is a model organism whose features include rapid growth, asexual reproduction (via propagules called gemmae), ease of performing genetic crosses, and established transformation methods (Ishizaki et al., 2016). *M. polymorpha* has a relatively small, sequenced genome (~280 Mb) that generally carries low genetic redundancy (Bowman et al., 2017). *M. polymorpha* belongs to possibly the earliest of land plant lineages (Wellman et al., 2003), and thus studies in *M. polymorpha* can provide evolutionary insights into plant hormones in land plant evolution.

Relatively little is known regarding the roles of ethylene in bryophytes. *M. polymorpha* is capable of producing ethylene, and its genome carries homologs of the entire ethylene signaling pathway as known in angiosperms (Li et al., 2020). Using CRISPR/Cas9 in *M. polymorpha*, Li et al. (2020) generated knockout alleles for two critical components in the ethylene signaling pathway, Mp*EIN3* and Mp*CTR1*. As shown in Li et al. (2020), MpEIN3 is the single ortholog of the EIN3 master transcription factor (Chao et al., 1997), and MpCTR1 is the single ortholog of the CTR1 protein kinase (Kieber et al., 1993), which signals immediately downstream of the ethylene receptors (Binder, 2020). CTR1 regulates EIN2, which leads to stabilization/accumulation of EIN3 (Binder, 2020). In *M. polymorpha*, Mp*EIN3* and Mp*CTR1* were found to have conserved roles as positive and negative regulators, respectively, of ethylene responses; that is, Mp*ein3* mutants exhibit ethylene insensitivity, while Mp*ctr1* mutants display constitutive responses to ethylene, like their orthologs in *A. thaliana* (Li et al., 2020). Ethylene responses in *M. polymorpha* include enhanced thallus growth, stimulation of gemmae cup formation, and increased gemmae dormancy (Li et al., 2020). Consequently, compared to the wild type, Mp*ctr1* plants are larger, have more gemma cups per thallus area, and have a higher proportion of dormant gemmae, whereas Mp*ein3* plants are smaller, have a lower density of gemma cups, and have a higher proportion of non-dormant gemmae (Li et al., 2020).

Here we carried out a genetic study to investigate whether ethylene signaling plays a protective role against abiotic stresses in *M. polymorpha*. We used the Mp*ctr1* and Mp*ein3* mutants to examine their ability to tolerate heat, salinity, nutrient deficiency, and continuous far-red light. We also examined whether abiotic stresses can induce ethylene biosynthesis in *M. polymorpha*. Our results demonstrate that ethylene is a stress hormone in *M. polymorpha*, providing protection against diverse abiotic stresses. Given that liverworts belong to one of the earliest lineages of land plants, these findings suggest that ethylene’s role as a stress hormone is at least as old as land colonization by plants.

## 2 Materials and methods

### 2.1 Plant material, culture conditions, and stress conditions

The *M. polymorpha* Australian (MEL accession) lines used in this study were previously described in Li et al. (2020): wild-type lines WT1 and WT2 (formerly named “WT3”), and three CRISPR/Cas9 knockout alleles of Mp*ein3* (L1, L5, L6) and three CRISPR/Cas9 knockout alleles of Mp*ctr1* (L1, L3, L4). Plants were maintained asexually by gemmae propagation. Individual gemmae were plated on solid growth medium in 60 mm x 15 mm Petri dishes with one gemma per dish unless otherwise indicated. All stresses were applied to gemmae starting from day 0 (the day of plating the gemmae), except for the treatments described in the Ethylene Production Assay section.

For non-stress conditions, plants were grown axenically from gemmae at 21 °C under continuous white light at 85-95 μmol m^-2^s^-1^ on a solid growth medium consisting of half-strength Gamborg’s B5 salts (Gamborg et al., 1968), pH 5.5, supplemented with 0.2% sucrose and 1% plant agar (Sigma). For heat stress, the plants were incubated in chambers at the stated temperatures. For salt stress, the medium was supplemented with the stated concentrations of NaCl. For nutrient deficiency, the Gamborg’s B5 salts were diluted to the stated concentrations (while keeping other medium contents the same). For far-red light treatment, continuous far-red light (730 nm) at 60 μmol m^-2^s^-1^ was added to the normal illumination (<0.15 R:FR ratio).

For ethylene biosynthesis measurements, plants were incubated in liquid growth medium in vials as described in the Ethylene Production Assay section.

### 2.2 Thallus ground cover area and density of gemma cups

Plant size was assessed by measuring the two-dimensional area using ImageJ (https://imagej.nih.gov/ij/). Plants were photographed using a digital camera (Nikon D3200). Using ImageJ, the color threshold was adjusted to cover the plants as much as possible with ‘RGB’ as the color space on the dark background setting. The wand (tracing) tool was used to measure the area after setting the pixels to a known length (in cm).

Relative ground cover area was determined by normalizing the average ground cover area of the control plants to 100%, and then calculating the average ground cover area of the treated plants as a percentage of the control.

The density of gemma cups was the number of gemma cups on a plant (counted by eye) divided by the ground cover area of that plant. For the relative density of gemma cups, the average density of gemma cups for the control plants was normalized to 100%, and then the average density of gemma cups for the treated plants was calculated as a percentage of the control.

These experiments were performed three times with similar results. For the 28 °C and 29 °C treatments we combined the data from the experimental replicates, but in all other cases, we show the data from one experiment rather than combining the data from replicate experiments.

### 2.3 Chlorophyll measurement

Extraction and measurement of chlorophyll content were performed as described in Welburn (1994) using dimethyl sulfoxide as the organic solvent. Thalli pieces were excised from 41-day old plants, weighed, and air dried before submerging them in 10 mL DMSO. Samples were then incubated at 60 °C for 1 hour with periodic shaking. Absorbance was determined at 649 nm and 665 nm using a GENESYS 10UV-Vis spectrophotometer. The chlorophyll experiment was performed once with *n* = 4-6 plants.

### 2.4 Ethylene production assay

Wild-type gemmalings (10-day old) were transferred from solid growth medium in Petri dishes to liquid medium in sterile 4-mL glass vials with septa-fitted airtight screw caps (Fisher Scientific). One plant was placed in each vial with only the rhizoids immersed in the liquid medium (with the dorsal side of the thallus facing upwards). The vials were placed in a growth chamber for 48 hours, after which 250 µL of gas from the headspace was removed through the septum using a syringe needle and injected into a gas chromatograph (Shimadzu GC-2010 PLUS) in order to measure ethylene. Following the ethylene measurements, the plants were weighed after briefly laying them on filter paper to absorb excess liquid.

For the control without treatment, the medium in the vial consisted of 300 µL of normal growth medium without agar. For heat stress, the vials were incubated at 29 °C. For salt stress, the liquid medium additionally contained 10 mM NaCl. For nutrient stress, the Gamborg’s B5 salts in the liquid medium were diluted 1/20. These experiments were performed three times with similar results. The data from one of these experiments is presented.

For far-red light treatment, the vials were laid on their sides for greater exposure to the far-red light. This experiment was performed once.

### 2.5 Statistical analysis

All probability analyses were performed using Prism (v.9.4.0, GraphPad). For comparisons between two groups, we used the two tailed t-test when the data were normally distributed and both groups had the same SD. For comparisons among multiple groups, we used one-way ANOVA with Tukey’s HSD *post hoc* test for populations that have normality of residuals and the same SD. When SD values were unequal (**Figure S2B**), we used the Brown–Forsythe and Welch ANOVA tests with Tamhane’s T2 *post hoc* test.

For the normalized graphs, we estimated the standard error of the mean (SEM) for the ratio between stress-treated mean value and the control mean value using the Taylor approximation of the variance for a ratio between two variables.

## 3 Results

### 3.1 Ethylene signaling plays a role in tolerance to abiotic stresses

#### 3.1.1 Heat stress

To determine the role of ethylene in heat stress tolerance, we assessed growth inhibition of two wild-type lines and the Mp*ein3* and Mp*ctr1* mutants (three alleles each) at elevated temperatures (27 °C to 30 °C) in comparison to growth at the normal temperature (21 °C). Gemmae were incubated at constant temperature starting at day 0 (the day gemmae were plated). After 14 days, we measured the “ground cover area” (i.e., the two-dimensional sizes) of the resulting plants. The 30 °C incubation was essentially lethal, as it severely inhibited gemmaling growth of all three genotypes and negatively impacted development, i.e., the plants were small and misshapen and did not produce gemmae (**Figures S1A, B**). At 30 °C, all three genotypes were inhibited to similar extents relative to their respective average sizes at the normal growth temperature (Figure S1C).

**Figure 1:**
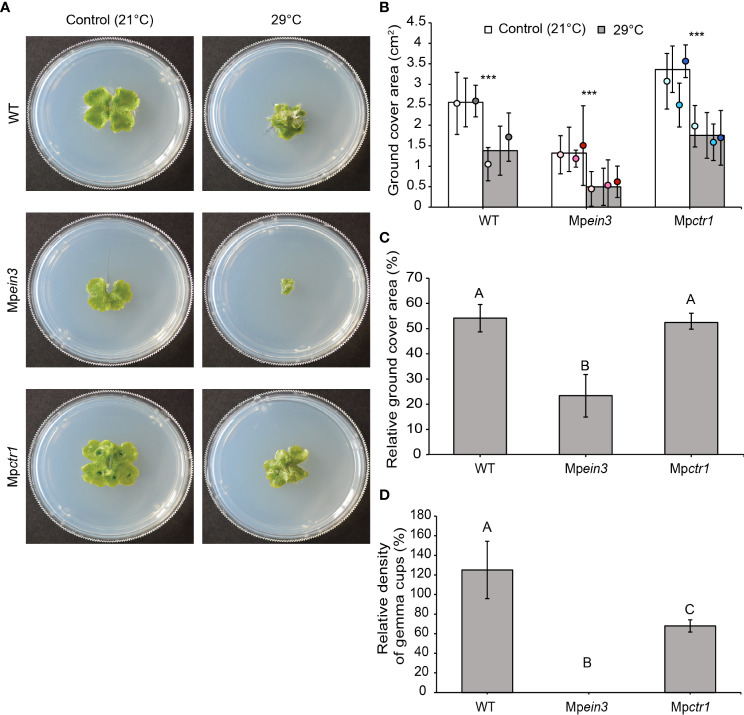
Mp*ein3* mutants are more sensitive to 29°C heat stress than Mp*ctr1* mutants or the wild type (WT). **(A)** Representative images of 14-day old WT, Mp*ein3*, and Mp*ctr1* plants grown at 21°C or 29°C. **(B)** Average size (ground cover area) of WT, Mp*ein3*, and Mp*ctr1* plants grown at 21°C and 29°C for 14 days. Bars show the overall mean ± SD. per genotype. Each data point represents the mean per WT line or mutant allele ± SD. For 21°C, *n*=24 WT (12 each of WT1 (white), WT2 (grey)); *n*=36 Mp*ein3* (11 L1 (light pink), 12 L5 (pink), 13 L6 (magenta) and *n*=36 Mp*ctr1* (12 L1 (light blue), 11 L3 (turquoise), 13 L4 (dark blue)). For 29°C, *n*=24 WT (12 each of WT1, WT2); *n*=32 Mp*ein3* (12 L1, 11 L5, 9 L6) and *n*=36 Mp*ctr1* (12 each of L1, L3, L4). *** = P<0.0001. P values were determined using a two-tailed t-test. **(C)** Relative ground cover area (based on the data in B) shown as a percentage of the corresponding control for each genotype. Bars show the mean ± SEM. Different letters indicate significant difference at P< 0.05, determined using one way ANOVA followed by Tukey’s HSD *post hoc* test (P<0.0001). **(D)** Relative density of gemma cups (for the plants treated with 29°C in B) shown as a percentage of the corresponding 21°C control for each genotype. (Absolute values are shown in **Table S1**.) Bars show the mean ± SEM. Different letters indicate significant difference at P< 0.05, determined using one way ANOVA followed by Tukey’s HSD *post hoc* test (P<0.0001).

Incubation at 29 °C exerted sub-lethal stress. After 14 days at 29 °C, all lines showed a more compact, circular thallus shape (e.g., **Figure 1A**). Mp*ctr1* plants were still larger than the wild type, but both the wild type and Mp*ctr1* were inhibited by roughly 47% relative to their respective average sizes (two-dimensional ground cover area) at 21 °C. In contrast, the Mp*ein3* ethylene-insensitive plants were inhibited by 77% relative to their average size at 21 °C (**Figures 1B, C**). We also assessed the number of gemma cups per thallus area at 29 °C versus 21 °C. Mp*ein3* produced no gemma cups at 29 °C (**Table S1**) and were more sensitive in terms of relative gemma cup density compared to Mp*ctr1* and the wild type (**Figure 1D**). These results indicated that ethylene hormone signaling provides some tolerance to heat stress at 29 °C.

Interestingly, incubation at 27 °C and 28 °C gave enhanced growth of all genotypes compared to their respective average sizes at 21 °C (**Figure S2**). Thus, 27 °C and 28 °C stimulate, rather than inhibit, gemmaling growth in *M. polymorpha*. At 28 °C, the relative growth of Mp*ein3* was greater than that of Mp*ctr1* (**Figure S2E**). Because this enhanced growth was observed even in the Mp*ein3* ethylene-insensitive mutant, the enhancement appeared to be independent of ethylene signaling.

#### 3.1.2 Salt stress

To examine the role of ethylene in tolerance to salinity, which exerts both ionic and osmotic stress, we first incubated gemmae of the wild type and Mp*ein3* mutant alleles for 14 days on growth medium supplemented with 0 mM, 25 mM, 75mM, and 150 mM NaCl. Images of representative 14-day old plants indicate that growth was inhibited by 25 mM and 75 mM NaCl, while 150 mM NaCl was essentially lethal to both genotypes (**Figure S3**). These results were consistent with the findings of Tanaka et al. (2018), who showed that growth of wild-type *M. polymorpha* is dramatically inhibited on medium containing 50 mM NaCl and completely inhibited on 250 mM NaCl.

**Figure 2:**
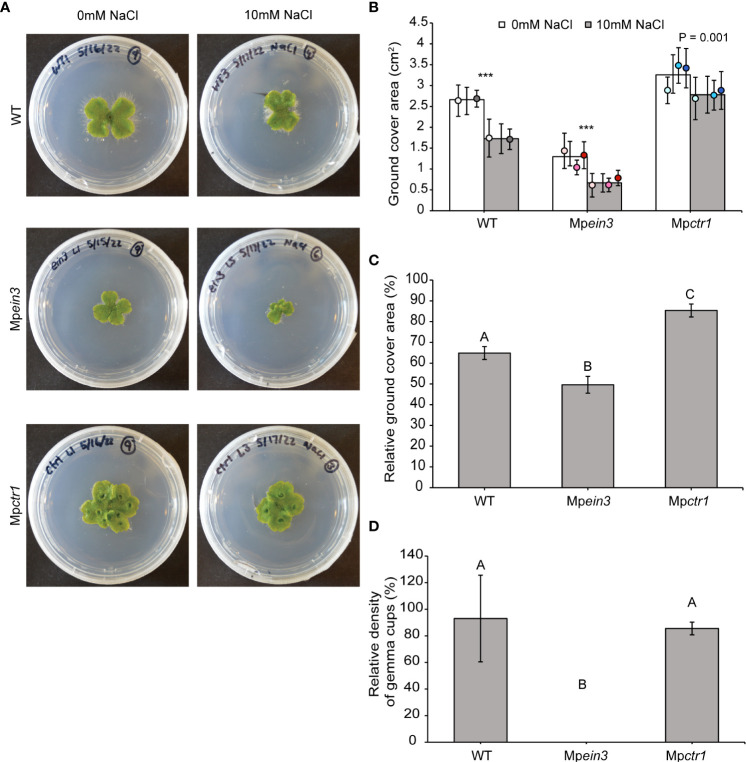
Mp*ein3* mutants are more sensitive to NaCl than Mp*ctr1* mutants or WT. **(A)** Representative images of 14-day old WT, Mp*ein3*, and Mp*ctr1* plants grown with or without 10 mM NaCl added to the growth medium. **(B)** Average size (ground cover area) of WT, Mp*ein3*, and Mp*ctr1* plants grown on medium containing 0 mM NaCl and 10 mM NaCl for 14 days. Bars show the overall mean ± SD per genotype. Each data point represents the mean per WT line or mutant allele ± SD. For 0 mM NaCl, *n*=24 WT (12 each of WT1 (white), WT2 (grey)); *n*=32 Mp*ein3* (12 L1 (light pink), 8 L5 (pink), 12 L6 (magenta)); *n*=35 Mp*ctr1* (12 L1 (light blue), 12 L3 (turquoise), 11 L4 (dark blue)). For 10 mM NaCl, *n*=24 WT (12 each of WT1, WT2); *n*=29 Mp*ein3* (9 L1, 11 L5, 9 L6); *n*=35 Mp*ctr1* (12 L1, 11 L3, 12 L4) *** = P<0.0001. P values were determined using a two-tailed t-test. **(C)** Relative ground cover area (based on the data in B) shown as a percentage of the corresponding control for each genotype. Bars show the mean ± SEM. Different letters indicate significant difference at P< 0.05, determined using one way ANOVA followed by Tukey’s HSD *post hoc* test (P<0.0001). **(D)** Relative density of gemma cups (for the plants treated with 10 mM NaCl in B) shown as a percentage of the corresponding 0 mM NaCl control for each genotype. (Absolute values are shown in **Table S1**.) Bars show the mean ± SEM. Different letters indicate significant difference at P< 0.05, determined using one way ANOVA followed by Tukey’s HSD *post hoc* test (P<0.0001).

On medium containing 10 mM NaCl, we found that growth of the wild type was only mildly inhibited (**Figure 2**). We then tested Mp*ein3* and Mp*ctr1* mutants on 10 mM NaCl. Interestingly, Mp*ctr1* plants fared better than the wild type, displaying no significant effects. In contrast, growth of Mp*ein3* plants was inhibited to a greater degree than the wild type (**Figures 2A–C**). At this dose, gemma cups still formed normally on wild-type and Mp*ctr1* plants, yet Mp*ein3* plants failed to produce gemma cups similar to what we observed at 29 °C (**Figure 2D** and **Table S1**). These results indicated that ethylene hormone signaling plays a role in tolerance to mild salt stress.

#### 3.1.3 Nutrient deficiency

**Figure 3:**
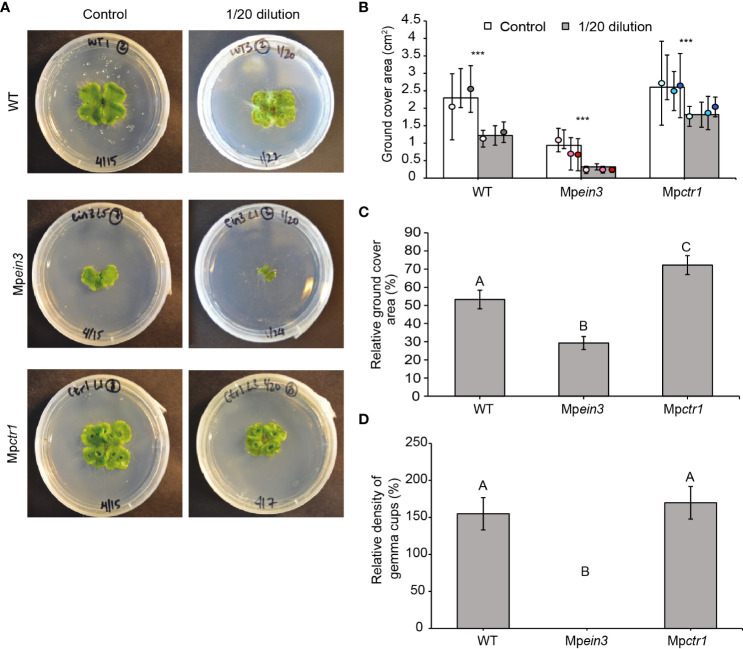
Mp*ein3* mutants are more sensitive to nutrient deficiency than Mp*ctr1* mutants or WT. **(A)** Representative images of 14-day old WT, Mp*ein3*, and Mp*ctr1* plants grown on regular medium (Control, containing a 2-fold dilution of Gamborg’s B5 salts) or containing a 1/20 dilution of Gamborg’s B5 salts. **(B)** Average size (ground cover area) of WT, Mp*ein3*, and Mp*ctr1* plants grown on regular medium and medium containing a 1/20 dilution of Gamborg’s B5 salts for 14 days. Bars show the overall mean ± SD per genotype. Each data point represents the mean per WT line or mutant allele ± SD. *n*=20 WT (10 each of WT1 (white), WT2 (grey)); *n*=30 Mp*ein3* (10 each of L1 (light pink), L3 (pink), L5 (magenta)); *n*=30 Mp*ctr1* (10 each of L1 (light blue), L3 (turquoise), L4 (dark blue)). *** = P<0.0001. P values were determined using a two-tailed t-test. **(C)** Relative ground cover area (based on the data in B) shown as a percentage of the corresponding control for each genotype. Bars show the mean ± SEM. Different letters indicate significant difference at P< 0.05, determined using one way ANOVA followed by Tukey’s HSD *post hoc* test (P<0.0001). **(D)** Relative density of gemma cups (for the plants grown on 1/20 Gamborg’s B5 salts in B) shown as a percentage of the corresponding control for each genotype. (Absolute values are shown in **Table S1**). Bars show the mean ± SEM. Different letters indicate significant difference at P< 0.05, determined using one way ANOVA followed by Tukey’s HSD *post hoc* test (P<0.0001).

To investigate the role of ethylene in resilience under nutrient deficiency, we incubated gemmae of the Mp*ein3* and Mp*ctr1* mutant alleles and the two wild-type lines on growth medium containing diluted Gamborg’s B5 salts. The normal growth medium consists of half-strength (2-fold dilution) of Gamborg’s B5 salts. On medium containing 1/30, 1/40, or 1/50 dilutions of Gamborg’s B5 salts, the growth of all three genotypes was severely inhibited (**Figure S4**). However, on a medium containing a 1/20 dilution of Gamborg’s B5 salts, growth of wild-type and Mp*ctr1* plants was only partially affected, whereas growth of Mp*ein3* plants was inhibited to a much greater extent, as shown by the relative growth inhibition (**Figures 3A–C**). These findings suggested that ethylene signaling provides a degree of protection against mild nutrient deficiency.

In Mp*ctr1* and wild-type plants, the 1/20 dilution of Gamborg’s B5 salts interestingly promoted the earlier formation of gemma cups compared to the control conditions and resulted in a higher number of gemma cups per ground cover area compared to the control (**Figure 3D** and **Table S1**). Gemma cups in control plants began to be visible on day 12 in Mp*ctr1* plants and day 14 in the wild type. With 1/20 dilution of Gamborg’s B5 salts, the Mp*ctr1* and wild-type plants showed gemma cup formation on days 10 and 12, respectively. In contrast, Mp*ein3* plants produced no gemmae cups under this stress, as seen for the 29 °C and 10 mM NaCl treatments.

At higher dilutions (1/30 and above), the center of the plants turned red, consistent with the induction of flavonoid production by nutrient deficiency in *M. polymorpha* (Albert et al., 2018).

#### 3.1.4 Far-red light

**Figure 4:**
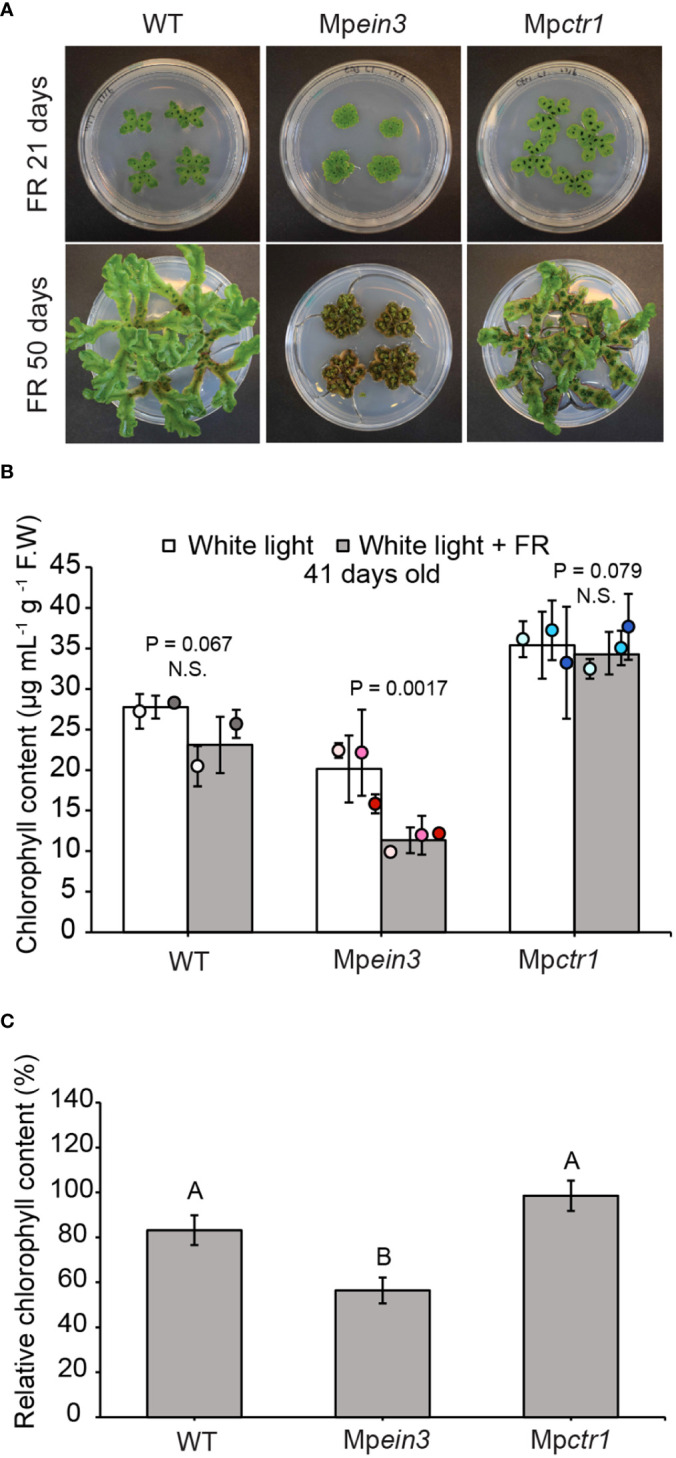
Mp*ein3* mutants are more sensitive to continuous far-red light than Mp*ctr1* mutants or WT. **(A)** Representative images of WT, Mp*ein3*, and Mp*ctr1* plants at 21 and 50 days of growth under continuous far-red light (in combination with normal white light). Each Petri dish (100 mm diameter) contains four plants. **(B)** Average chlorophyll content of 41-day old WT, Mp*ein3*, and Mp*ctr1* plants grown under continuous white light (control) with and without continuous far-red light. Bars show the overall mean ± SD per genotype. Each data point represents the mean per WT line or mutant allele ± SD. *n*=4 WT (2 each of WT1 (white), WT2 (grey)); *n*=6 Mp*ein3* (2 each of L1 (light pink), L3 (pink), L5 (magenta)); *n*=6 Mp*ctr1* (2 each of L1 (light blue), L3 (turquoise), L4 (dark blue)). P values were determined using a two-tailed t-test. N.S., not significant. **(C)** Relative chlorophyll content (based on the data in B) shown as a percentage of the corresponding control for each genotype. Bars show the mean ± SEM. Different letters indicate significant difference at P< 0.05, determined using one way ANOVA followed by Tukey’s HSD *post hoc* test (P<0.0001) (P value for WT versus Mp*ctr1* = 0.051).

In angiosperms, a high level of continuous far-red light relative to red light exerts various effects on plant morphology and, in some cases, results in longer, narrower leaves (Tan et al., 2022). Here we incubated gemmae of Mp*ein3* and Mp*ctr1* mutants and the wild type under normal white light supplemented with or without continuous far-red light (60 μmol m^-2^s^-1^) (<0.15 R:FR ratio). Between 21 days and 50 days of treatment, the wild type and Mp*ctr1* plants appeared to have longer, narrower thalli that were more highly branched (**Figure 4A**). In contrast, Mp*ein3* mutants displayed a highly compact morphology with short, rounded thalli (**Figure 4A**). By 50 days under the far-red light treatment, the Mp*ein3* thalli had largely turned brown, whereas the wild type and Mp*ctr1* plants remained mostly green (**Figure 4A**).

Measurement of chlorophyll content in 41-day old plants with and without far-red light treatment showed that wild-type and Mp*ctr1* plants had a higher level of chlorophyll that was not significantly reduced by far-red light treatment, whereas Mp*ein3* plants had a lower level of chlorophyll that was reduced by 44%. (**Figures 4B, C**). These results suggested that ethylene signaling plays a role in far-red light tolerance in *M. polymorpha*.

### 3.2 Ethylene production is enhanced in response to abiotic stresses

**Figure 5:**
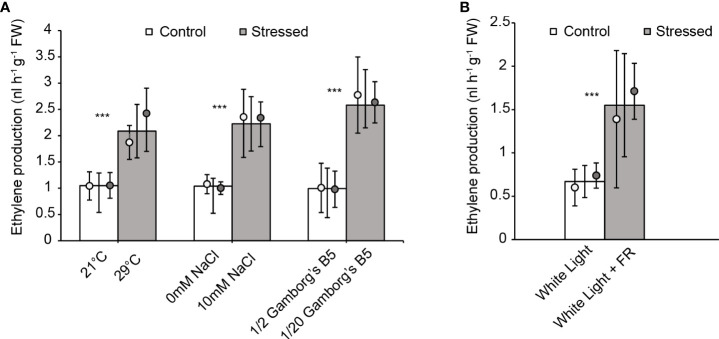
Abiotic stress enhances ethylene production in *M. polymorpha*. Ethylene production was measured in 10-day old wild-type plants after 48 hr incubation under **(A)** elevated temperature (29°C), salinity (10 mM NaCl), or nutrient deficiency (1/20 dilution of Gamborg’s B5 salts) (with vials incubated upright) or **(B)** under white light plus far-red light (with vials incubated on their sides). Bars show the overall mean ± SD per genotype. Each data point represents the mean per wild-type line. *n*=10 (5 each of WT1 (white), WT2 (grey)). *** = P<0.0001. P values were determined using a two-tailed t-test.

Given that ethylene signaling plays a role in protection against the above stresses, we hypothesized that abiotic stress would induce ethylene biosynthesis. To test this, we treated 10-day old wild-type plants separately with and without high temperature (29 °C), salt (10 mM NaCl), nutrient deficiency (20-fold dilution of Gamborg’s B5 medium), or continuous far-red light and then measured ethylene production. Plants were treated individually in an airtight vial on liquid medium, and after 48 hours of treatment, we measured the concentration of ethylene in the headspace by gas chromatography. All three stress conditions (29 °C, 10 mM NaCl, and nutrient deficiency) showed an increase in ethylene levels compared to the controls without stress treatment (**Figure 5A**). While not necessarily considered an abiotic stress, the treatment with continuous far-red light also led to ethylene production (**Figure 5B**). These results show that environmental stresses can induce ethylene biosynthesis in *M. polymorpha*, consistent with our data indicating that ethylene signaling promotes resilience under abiotic stress.

## 4 Discussion

Our results indicate that ethylene serves as a stress hormone in the liverwort *M. polymorpha*. Specifically, we found that ethylene biosynthesis increases when *M. polymorpha* is under abiotic stress, and that ethylene signaling provides a degree of protection against abiotic stress. We base these conclusions on mild or sub-lethal doses of abiotic stresses that inhibit *M. polymorpha* growth: heat (29 °C), salt (10 mM NaCl), nutrient deficiency (20-fold dilution of Gamborg’s B5 salts), and continuous far-red light. Under these conditions, the growth of Mp*ctr1* constitutive ethylene response mutants and wild-type plants was only partially inhibited, whereas growth of Mp*ein3* ethylene-insensitive mutants was substantially inhibited, indicating that ethylene signaling through MpCTR1 and MpEIN3 plays a role in stress resilience. In *A. thaliana*, AtEIN3 activates several downstream transcription factors to initiate basal thermotolerance (Huang et al., 2021), and the stabilization of AtEIN3 under salt stress prevents ROS accumulation leading to salt tolerance (Peng et al., 2014). Similarly, AtEIN3 plays a role in ethylene-mediated responses to nutrient deficiency (García et al., 2021). Our findings suggest that ethylene signaling may have conserved functions in abiotic stress signaling in *M. polymorpha*.

Under heat and nutrient deficiency, the wild type displayed essentially the same extent of stress tolerance (i.e., growth) as the Mp*ctr1* mutants. This observation is expected because these abiotic stresses enhance ethylene production in the wild type (**Figure 5**), and the concomitant increase in ethylene signaling likely results in a response that equals the constitutive ethylene response conferred by the Mp*ctr1* mutant alleles. Under mild salt stress, the Mp*ctr1* mutants actually displayed greater resilience than the wild type. While the underlying mechanisms for this result are unknown, this illustrates the opposing roles of Mp*CTR1* and Mp*EIN3* as negative and positive regulators of abiotic stress responses, respectively.

Interestingly, the growth of *M. polymorpha* was enhanced at 27 °C and 28 °C, even in the Mp*ein3* mutants. This faster growth might result from biological processes being sped up at higher temperatures. We speculate that prolonged exposure to 27 °C or 28 °C (beyond our experimental timeframe) might eventually be detrimental to growth. Given that constant exposure to 30 °C severely limited growth of all genotypes, while constant exposure to 27 °C or 28 °C enhanced growth of all genotypes, ethylene appears to play a role within a narrow window of heat stress in *M. polymorpha* under the growth conditions we used. We also observed that nutrient deficiency enhanced production of gemma cups in both the wild type and the Mp*ctr1* mutants.

We also examined the role of ethylene signaling in *M. polymorpha* in response to continuous far-red light (<0.15 R:FR ratio). Far-red light evokes several physiological responses but is not necessarily considered an abiotic stress. In fact, the removal or reduction of phytochrome activity has been shown to enhance resilience under stress in both *A. thaliana* (Yang et al., 2016) and tomato (Cao et al., 2018). In the laboratory, far-red light is used to induce sexual reproduction in *M. polymorpha* (Ishizaki et al., 2016). Far-red light, which is enriched beneath a canopy, can lead to extension growth as a shade avoidance response (Pierik et al., 2009; Tan et al., 2022). When treated with far-red light, we observed morphological changes (elongation, narrowing, and more branching) of thalli in the wild type and Mp*ctr1*. These changes conceivably comprise a shade avoidance response. Given that the Mp*ein3* mutants lacked this response to far-red light, the response appears to involve ethylene signaling and is perhaps related to the role of ethylene in promoting plant growth in *M. polymorpha* (Li et al., 2020). Such growth could occur through increased cell expansion and cell division, as shown for epidermal cells of ethylene-treated gemmae (Li et al., 2020). Alternatively, there could be a direct interaction between the phytochrome signaling pathway and EIN3 as in *A. thaliana* (Shi et al., 2016).

All four of the stresses we tested enhanced ethylene biosynthesis. While *M. polymorpha* clearly synthesizes ethylene, we note that the ethylene biosynthesis pathway has yet to be identified in *M. polymorpha* and in non-seed plants in general (Li et al., 2022). In angiosperms, the ethylene biosynthesis pathway is well known and involves the conversion of 1-aminocyclopropane-1-carboxylic acid (ACC) to ethylene by the enzyme ACC oxidase. Non-seed plants, however, lack the genes for ACC oxidase (Li et al., 2018). At best, ACC is a weak ethylene precursor in *M. polymorpha* (Li et al., 2020).

In angiosperms, stress-induced ethylene production and signaling lead to the activation of multiple stress-responsive pathways, often *via* Ethylene Response Factor (ERF) transcription factors (Debbarma et al., 2019). Abiotic stressors such as salinity, drought, heat, flooding, and nutrient stress all lead to a characteristic burst in ROS levels (Choudbury et al., 2017) and a drop in photosynthesis (Sharma et al., 2020). Drought, salt and heat stress also activate osmotic responses which entail the production of heat-shock proteins, osmolytic proteins and osmolytes (Hasanuzzaman et al., 2013; van Zelm et al., 2020), while nutrient and salinity stress trigger the synthesis of ion transporters (van Zelm et al., 2020). Whether these stress responses are activated in *M. polymorpha* in an ethylene-dependent manner remains to be studied, but initial genomic and transcriptomic analyses of salt- and heat-stressed *M. polymorpha* plants revealed that genes involved in oxidative stress (ROS metabolism) were conserved and enriched (Tanaka et al., 2018; Marchetti et al., 2021). Additionally, transcriptional changes in ethylene signaling genes have been observed under conditions of phosphate starvation in *M. polymorpha* (Rico-Resendiz et al., 2021), although the phenotypic consequences remain unknown.

Our findings in *M. polymorpha* indicate that this stress-related role of ethylene production and signaling likely predated the divergence of the bryophytes at least 450 million years ago from the land plant lineage. The role of ethylene as a stress hormone in bryophytes was previously reported for an ethylene-mediated submergence (flooding) response in the moss *Physcomitrella patens* (Yasumura et al., 2012). During flooding, wild-type *P. patens* plants alter their developmental programming towards colonizing behavior, and this escape strategy is dependent on ethylene signaling, given that an ethylene-insensitive receptor mutant was found to be defective in this response (Yasumura et al., 2012). Key components of the ethylene signaling pathway have been shown to be functionally conserved in the Charophycean alga *Spirogyra pratensis* (Ju et al., 2015), and therefore it is possible that ethylene signaling could have facilitated stress resilience prior to the evolutionary transition to land. Connections between abiotic stress and ethylene signaling have been observed in *S. pratensis* without detectable increases in ethylene biosynthesis under various stress conditions (Van de Poel et al., 2016). Ethylene might have played a role in facilitating the transition from an aquatic habitat to a terrestrial habitat to help plants deal with the environmental stresses of life on land, such as drought, heat, and UV radiation. Our findings demonstrate that ethylene biosynthesis and signaling play a role in abiotic stress resilience in *M. polymorpha*, indicating that ethylene has likely been conserved as a stress hormone for at least 450 million years of plant evolution.

While we have shown that ethylene plays a role in responses to abiotic stress in *M. polymorpha*, an interplay with other signaling pathways is likely to be involved (Verma et al., 2016; Dubois et al., 2018). Charophycean algae also contain stress-related pathways that enable them to adapt to changing environmental conditions (Holzinger et al., 2014; Hori et al., 2014; de Vries et al., 2018; de Vries et al., 2020). In *M. polymorpha*, tolerance to heat stress is linked to abscisic acid, jasmonic acid, and auxin signaling (Marchetti et al., 2021), and coordination of growth and oxidative stress response in *M. polymorpha* involves the conserved DELLA transcription factor (Hernández-García et al., 2021). Unraveling these stress response networks and their interaction with ethylene will be valuable for inferring which pathways may have been present in the ancestor of early land plants and for developing ways to mitigate the detrimental effects of abiotic stresses that are increasingly prevalent due to climate change.

## Data availability statement

The original contributions presented in the study are included in the article/**Supplementary Material**. Further inquiries can be directed to the corresponding author.

## Author contributions

CC, PB, and LS conceived and designed the experiments. LS and DE carried out heat stress experiments. LS and DL carried out salt stress experiments. PB carried out nutrient deficiency experiments, chlorophyll measurements, and ethylene production assays and prepared the figures. DL carried out far-red light and salt stress experiments in BV’s lab. CC and PB wrote the manuscript with assistance from all of the authors. All authors contributed to the article and approved the submitted version.

## Funding

This work was supported by an NSF grant (MCB-1714993) to CC and a VLAIO grant (HBC.2020.3207) and KU Leuven grant (C14/18/056) to BV. CC is supported in part by the Maryland Agricultural Experiment Station.

## Acknowledgments

We thank Tom Dierschke (Monash University, Australia) in Dr. John L. Bowman’s laboratory for sharing his observation that the Mp*ein3* mutants are hypersensitive to far-red light. We thank Dr. Jianhong Chang for helpful discussions on statistical analyses. We also thank Husan Turdiev (Chang lab) for assistance with photographing *M. polymorpha* and Stijn Roden (Van de Poel lab) for shipping *M. polymorpha* lines. We thank members of the Chang lab for comments on the manuscript.

## Conflict of interest

The authors declare that the research was conducted in the absence of any commercial or financial relationships that could be construed as a potential conflict of interest.

## Publisher’s note

All claims expressed in this article are solely those of the authors and do not necessarily represent those of their affiliated organizations, or those of the publisher, the editors and the reviewers. Any product that may be evaluated in this article, or claim that may be made by its manufacturer, is not guaranteed or endorsed by the publisher.
