# Supplementary material for: The plant hormone ethylene promotes abiotic stress tolerance in the liverwort Marchantia polymorpha
Source: Front Plant Sci. 2022 Oct 18;13:998267. doi: 10.3389/fpls.2022.998267 (PMC9632724; doi:10.3389/fpls.2022.998267)
Supplement: Supplementary file 1 [file DataSheet_1.pdf]

Supplementary Material

Supplementary Figure 1

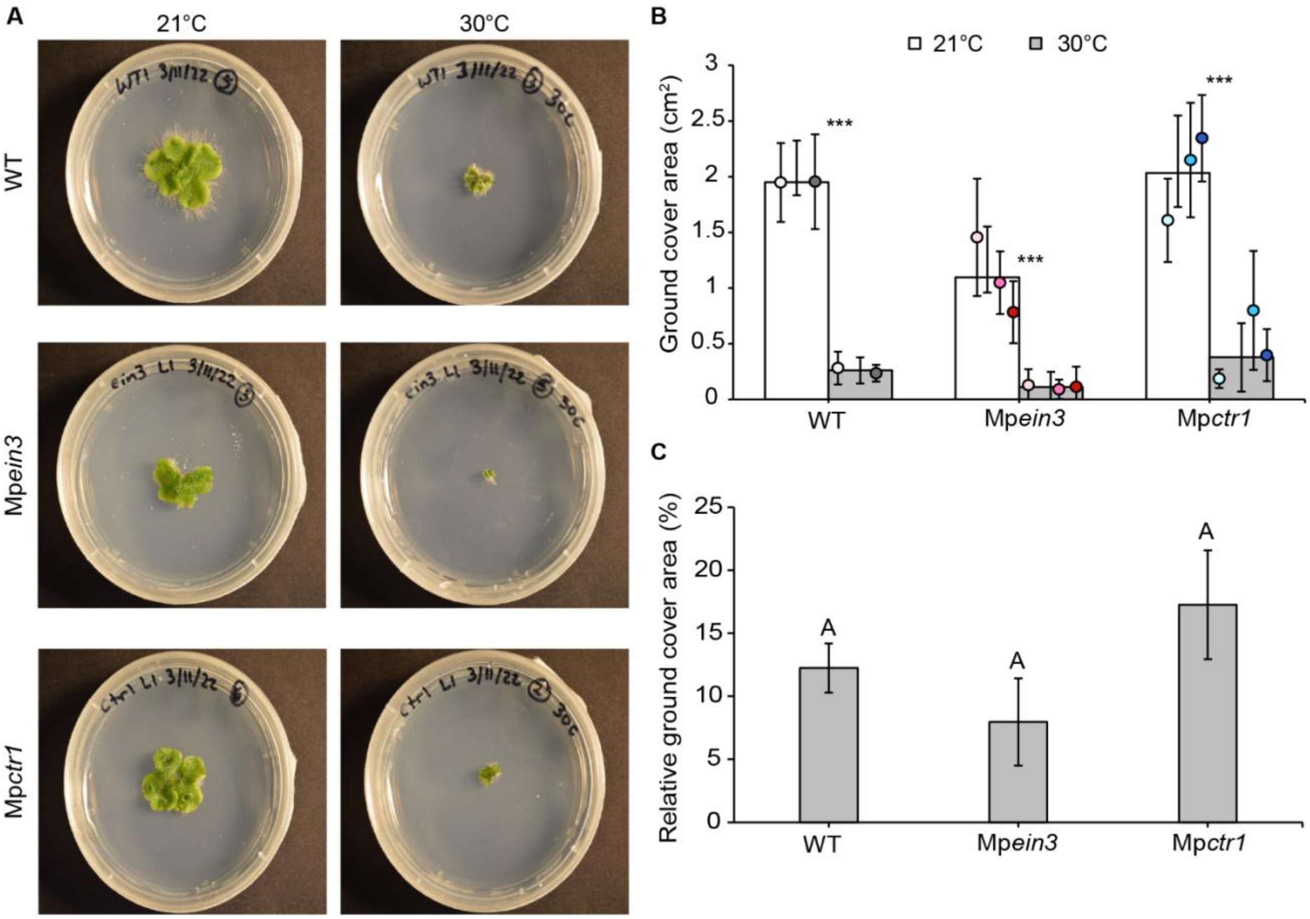

Supplementary Figure 1. Incubation at 30 °C severely inhibits growth of *M. polymorpha.* (A) Representative images of 14-day old WT, Mp*ein3*, and Mp*ctr1*plants grown at 21 °C and 30 °C. **(B)** Average size (ground cover area) of WT, Mp*ein3*, and Mp*ctr1* plants grown at 21 °C and 30 °C for 14 days. Bars show the mean ± SD per genotype. Each data point represents the mean per WT line or mutant allele ± SD. At 21 °C, *n*=12 WT (6 each of WT1 (white), WT2 (grey)); *n*=18 Mp*ein3* (6 each

of L1 (light pink), L3 (pink), L5 (magenta)); *n*=18 Mp*ctr1* (6 each of L1 (light blue), L3 (turquoise), L4 (dark blue)). At 30 °C, *n*=11 WT (6 of WT1, 5 of WT2); *n*=14 Mp*ein3* (5 L1, 4 L3, 5 L5); *n*=13 Mp*ctr1* (5 L1, 2 L3, 6 L4). *** = P< 0.0001. P values were determined using a two-tailed t-test. **(C)** Relative ground cover area (based on the data in **B**) shown as a percentage of the corresponding control for each genotype. Bars show the mean ± SEM. The same letters indicate no significant difference at P< 0.05, determined using one way ANOVA followed by Tukey's HSD post hoc test (P= 0.1063).

Supplementary Figure 2

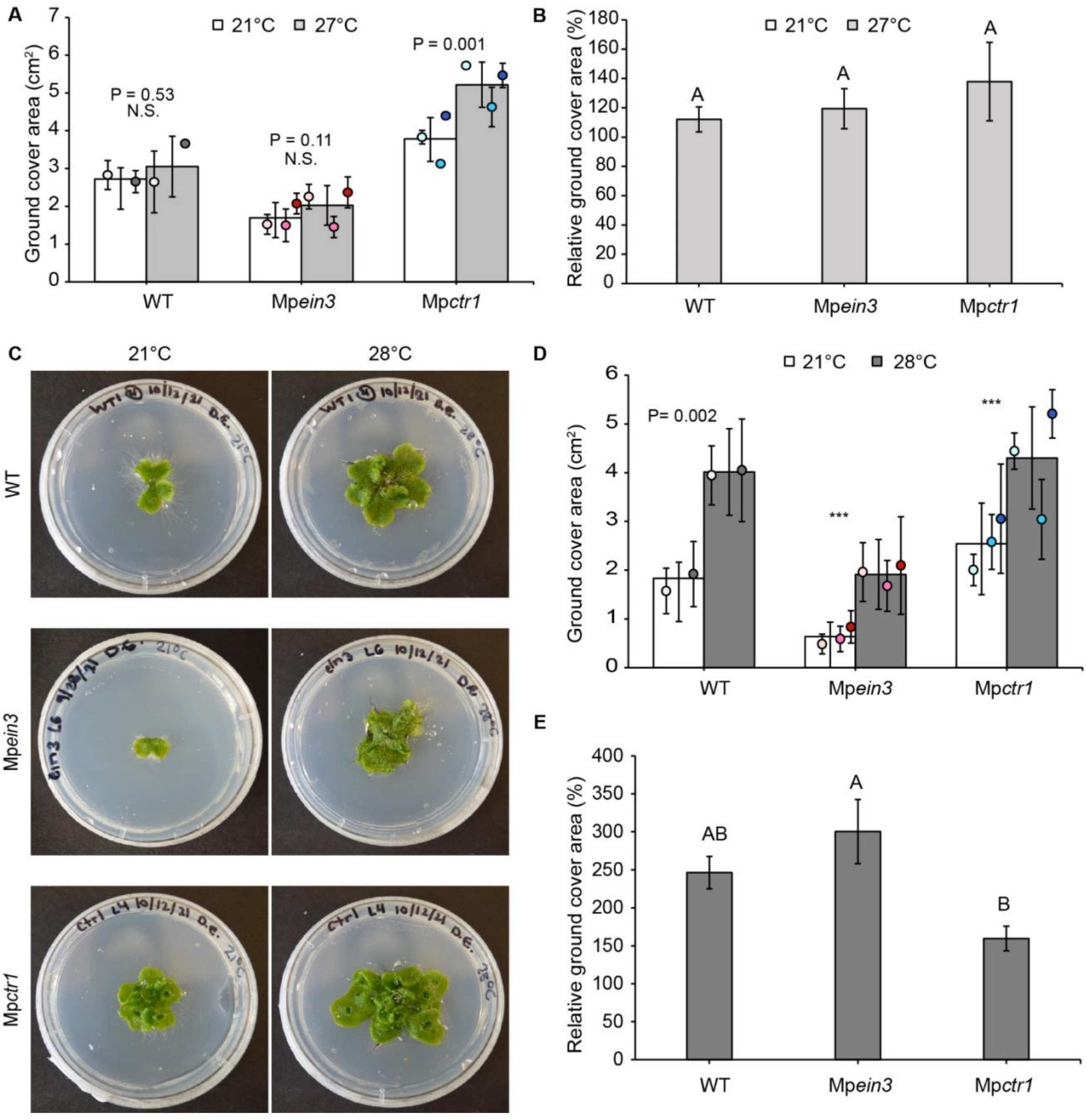

Supplementary Figure 2. Incubation at 27 °C or 28 °C stimulates growth of *M. polymorpha*. (A) Average size (ground cover area) of 14-day old WT, Mp*ein3*, and Mp*ctr1* grown at 21 °C and 27 °C for 14 days. Bars show the mean ± SD per genotype. Each data point represents the mean per WT

line or mutant allele ± SD. At 21°C, *n*=5 WT (2 WT1 (white), 3 WT2 (grey)); *n*=9 Mp*ein3* (3 each of L1 (light pink), L5 (pink), L6 (magenta)) and *n*=9 Mp*ctr1* (3 each of L1 (light blue), L3 (turquoise), L4 (dark blue)). At 27 °C, *n*=5 WT (2 WT1, 3 WT2); *n*=9 Mp*ein3* (3 each of L1, L5, L6) and *n*=8 Mp*ctr1* (2 L1, 3 L3, 3 L4). P values were determined by a two-tailed t-test. N.S. = not significant. **(B)** Relative ground cover area (based on the data in **A**) shown as a percentage of the corresponding control for each genotype. Bars show the mean ± SEM. The same letters above the bars indicate no significant difference at $P < 0.05$, determined using Brown–Forsythe and Welch ANOVA followed by Tamhane's T2 post hoc test ($P > 0.99$). **(C)** Representative images of 14-day old WT, Mp*ein3*, and Mp*ctr1* plants grown at 21 °C or 28 °C. **(D)** Average size (ground cover area) of WT, Mp*ein3*, and Mp*ctr1* plants grown at 21 °C and 28 °C for 14 days. Bars show the mean ± SD per genotype. Each data point represents the mean per WT line or mutant allele ± SD. At 21°C, n=8 WT (2 WT1, 6 WT2); n= 18 Mp*ein3* (6 each of L1, L5, L6), n= 18 Mp*ctr1* (6 each of L1, L3, L4). At 28 °C, n=9 WT (3 WT1, 6 WT2); n= 18 Mp*ein3* (6 each of L1, L5, L6), n= 17 Mp*ctr1* (6 L1, 5 L3, 6 L4). *** = $P < 0.0001$. P values were determined using a two-tailed t-test. **(E)** Relative ground cover area (based on the data in **D**) shown as a percentage of the corresponding control for each genotype. Bars show the mean ± SEM. Different letters indicate significant difference at $P < 0.05$, determined using one way ANOVA followed by Tukey's HSD post hoc test ($P < 0.0001$).

Supplementary Figure 3

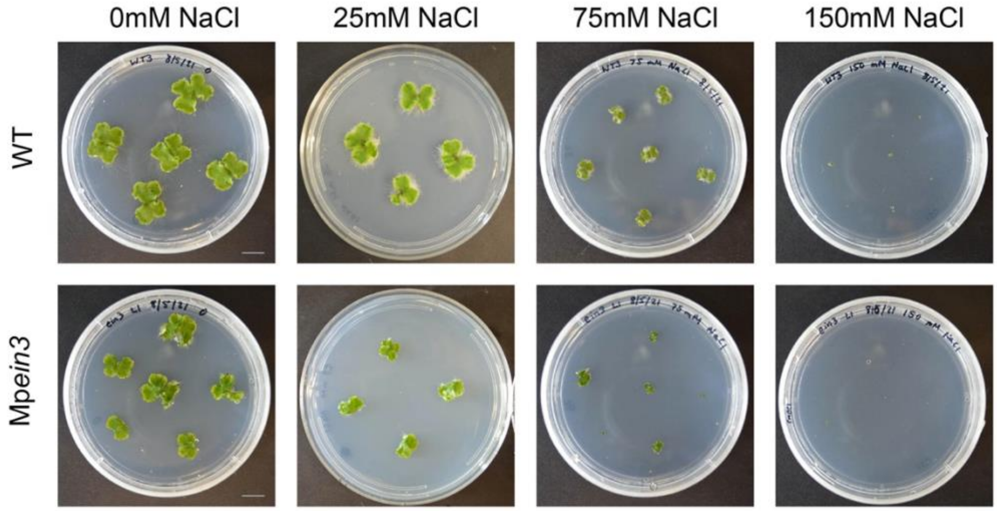

Supplementary Figure 3. Growth of wild-type and *Mpein3* plants on medium containing NaCl. Representative images of 14-day old WT and Mp*ein3* on medium supplemented with 0, 25, 75, and 150 mM NaCl. Petri dishes are 100 mm in diameter with 4-6 gemmae plated per dish.

Supplementary Figure 4

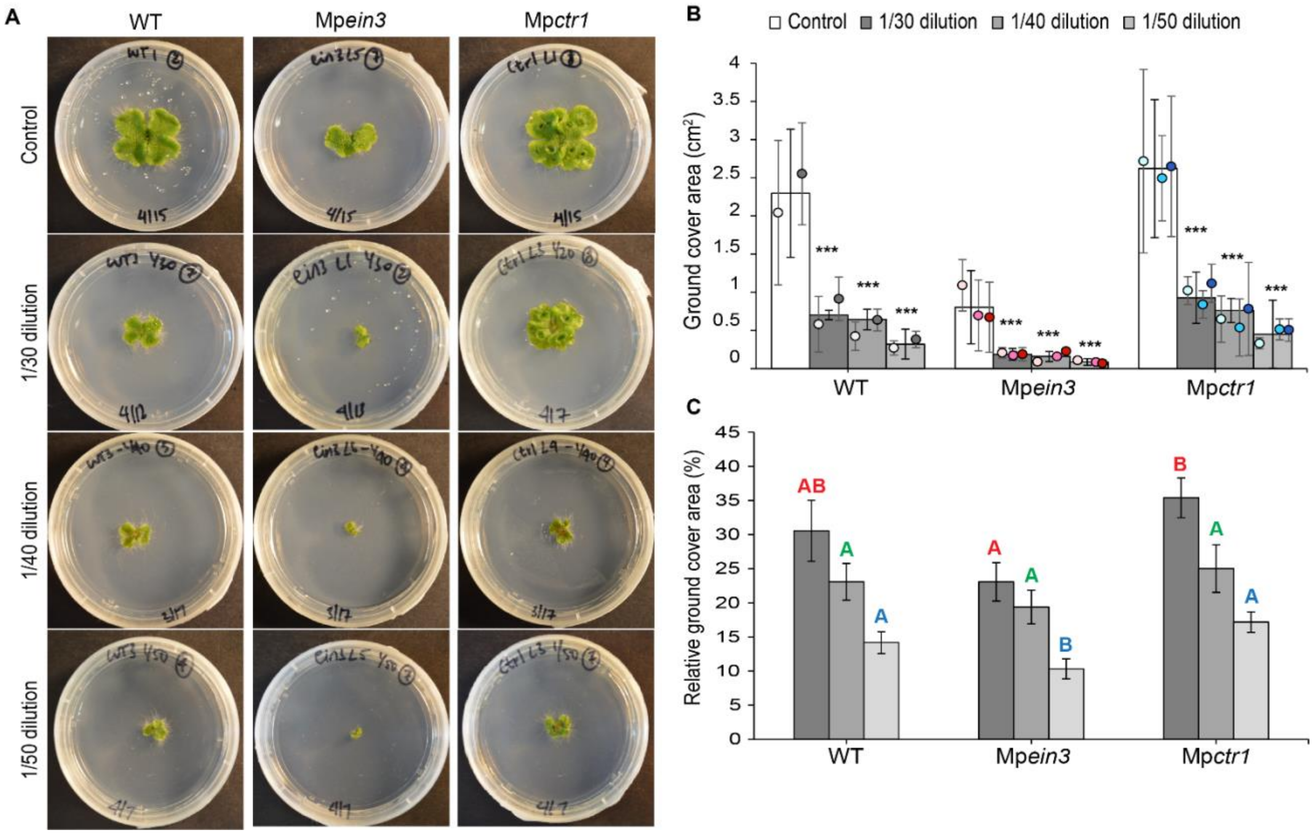

Supplementary Figure 4. Growth of wild type, Mp*ein3*, and Mp*ctr1* in response to nutrient deficiency. (A) Representative images of 14-day old WT, Mp*ein3*, and Mp*ctr1* plants grown on regular medium containing a 2-fold dilution of Gamborg's B5 salts (Control), or containing 1/30, 1/40, or 1/50 dilutions of Gamborg's B5 salts. **(B)** Average size (ground cover area) of WT, Mp*ein3*, and Mp*ctr1* plants grown on regular medium and medium containing a 1/20 dilution, 1/30 dilution, 1/40 dilution and 1/50 dilution of Gamborg's B5 salts for 14 days. Bars show the mean ± SD per genotype. Each data point represents the mean per WT line or mutant allele ± SD. For the control, *n*=20 WT (10 each of WT1 (white), WT2 (grey)); *n*=30 Mp*ein3* (10 each of L1 (light pink), L3 (pink), L5 (magenta)); *n*=30 Mp*ctr1* (10 each of L1 (light blue), L3 (turquoise), L4 (dark blue)). For the 1/30 dilution, *n*=19 WT (10 WT1, 9 WT2); *n*=29 Mp*ein3* (10 L1, 10 L3, 9 L5); *n*=28 Mp*ctr1* (10 L1, 9 L3, 9 L4). For the 1/40 dilution, *n*=20 WT (10 each of WT1, WT2); *n*=30 Mp*ein3* (10 each of L1, L3, L5); *n*=30 Mp*ctr1* (10 each of L1, L3, L4). For the 1/50 dilution , *n*=20 WT (10 each of WT1, WT2); *n*=29 Mp*ein3* (10 L1, 10 L3, and 9 L5); *n*=30 Mp*ctr1* (10 each of L1, L3, L4). *** = $P<0.0001$. P values were determined using a two-tailed t-test comparing the sample mean to the respective control mean. **(C)** Relative ground cover area (based on the data in **B**) shown as a percentage of the corresponding control for each genotype. Bars show the mean ± SEM. Different letters in the same color above the bars (red for 1/30 dilution; green for 1/40 dilution; blue for 1/50 dilution) represent significance at $P< 0.05$ determined by one way ANOVA followed by Tukey's

HSD post hoc test (for 1/30 dilution, $P<0.001$; for 1/40 dilution, $P=0.206$; for 1/50 dilution, $P<0.0001$). There is no significant difference for the 1/40 dilution (green).

Supplementary Table 1. Density of Gemma Cups*

	Mean # gemma cups per thallus ground cover area +/- SD		
Treatment	WT	Mp*ein3*	Mp*ctr1*
21 ^{o}C	1.02 +/- 0.72	0.84 +/- 0.91	2.81 +/- 0.80
29 ^{o}C	1.51 +/- 1.00	0.0 +/- 0.0	1.91 +/- 0.92
	P = 0.06 (N.S.) †	P < 0.01 †	P < 0.0001 †
0 mM NaCl	0.43 +/- 0.54	0.47 +/- 0.63	2.46 +/- 0.52
10 mM NaCl	0.40 +/- 0.465	0.0 +/- 0.0	2.10 +/- 0.53
	P = 0.84 (N.S.) †	P < 0.001 †	P < 0.01 †
1/2 Gamborg's B5	1.41 +/- 1.006	0.77 +/- 0.62	3.09 +/- 2.00
1/20 Gamborg's B5	2.49 +/- 0.86	0 +/- 0	5.24 +/- 2.99
	P < 0.001 †	P < 0.0001 †	P = 0.001 †

*Data are from the same plants analyzed in **Figures 1B** (heat), **2B** (salt) and **3B** (nutrient deficiency).
† P values were determined by the two-tailed t-test comparing the means of the control and stress treatments per genotype. N.S. = not significant.
